# Camel Prion Disease, Tataouine, Tunisia, 2019–2021

**DOI:** 10.3201/eid3208.251474

**Published:** 2026-08

**Authors:** Abdelkader Amara, Michele Angelo Di Bari, Kéfia Elmehatli, Rosalia Bruno, Rihab Andolsi, Barbara Chiappini, Ilaria Vanni, Elena Esposito, Geraldina Riccardi, Obaid Allah Ben Abid, Stefano Marcon, Atef Malek, Boubaker Ben Smida, Haykel Kessa, Walid Chandoul, Mariem Handous, Roukaya Khorchani, Romolo Nonno, Malek Zrelli, Umberto Agrimi, Gabriele Vaccari, Laura Pirisinu

**Affiliations:** Université Mannouba Tunisie, École Nationale de Médecine Vétérinaire, Sidi Thabet, Tunisia (A. Amara, R. Andolsi, A. Malek); Istituto Superiore di Sanità, Rome, Italy (M.A. Di Bari, R. Bruno, B. Chiappini, I. Vanni, E. Esposito, G. Riccardi, O.A. Ben Abid, S. Marcon, R. Nonno, U. Agrimi, G. Vaccari, L. Pirisinu); Arrondissement de Production Animale de Siliana, Siliana, Tunisia (K. Emehatli); Regional Delegation for Agricultural Development in Tataouine, Tataouine, Tunisia (B. Ben Smida); Arrondissement de Production Animale de Sousse, Sousse, Tunisia (H. Kessa); Regional Delegation for Agriculture in Medenine, Medenine, Tunisia (W. Chandoul); Institut Pasteur de Tunisie, Tunis, Tunisia (M. Handous); Direction Générale des Services Vétérinaires, Ministère de l’Agriculture, Tunis (R. Khorchani) Ministry of Agriculture, Tunis (M. Zrelli)

**Keywords:** prions, camel prion disease, CPrD, dromedary camel, Camelus dromedarius, animal prion disease, scrapie, bovine spongiform encephalopathy, BSE, Tunisia

## Abstract

We report 6 cases of camel prion disease in dromedaries in Tunisia, confirming widespread occurrence of the disease in North Africa. Affected animals showed neurologic signs and disease-associated prion protein accumulation in brain and lymphoid tissues. These findings highlight the importance of active surveillance and investigation of the epidemiology, transmission, and public health implications of this disease.

In 2018, a novel animal prion disease was identified in Algeria, termed camel prion disease (CPrD), affecting a previously unreported host species, the dromedary camel (*Camelus dromedarius*) (*1*). After identification of CPrD in Algeria, an epidemiologic surveillance network was set up in Tunisia to monitor neurologic diseases in dromedaries. We describe results of investigations of suspected cases and report the detection of 6 CPrD cases.

## The Study

Tunisia hosts ≈57,000 dromedaries, 75% of them in the south. Accordingly, investigations focused on Tataouine, the southernmost governorate. Over 3 years, we identified 8 dromedary camels showing neurologic and behavioral signs consistent with CPrD (*1*), raising suspicion of prion disease (Table).

**Table:** Suspect cases of camel prion disease, Tunisia, 2019–2021*

Animal ID	Age, y	Origin	Clinical signs	PrP genotype	Brain			Lymph node IHC
					WB	H&E	IHC	
P81/9	12	Dhaher Tataouine	Disorientation, impaired spatial orientation, fugue-like behavior	WT/WT	+	–	+	+
P81/13	3	Dhaher Tataouine	Nervous disorder referred as “Medhbouba”	WT/WT	+	+‡	+	NA
P81/14	3	Remada of Tataouine	Nervous disorder referred as “Medhbouba”	WT/WT	+	+‡	+	NA
P81/15	20	Remada of Tataouine	Suppurative mastitis, paresis of the left hind limb	WT/WT	–	–	–	NA
P81/16	15	Algeria, grazing in Remada of Tataouine	Nervous disorder referred as “Medhbouba”	WT/WT	+	+	+	+
P81/17	5	Remada of Tataouine	Behavioral changes, teeth grinding	WT/WT	+	–	+	+
P81/64†	25	Sousse	Anorexia, mild ataxia, muscle tremors, paddling movements	ND	–	–	–	NA
P81/65	3	Tataouine	Trembling, loss of appetite	WT/WT	+	+	+	+

*ND, not determined; H&E, hematoxylin and eosin staining; ID, identification number; IHC, immunohistochemistry; NA, not available; WB, western blot; WT/WT, homozygous wild-type allele (GenBank accession no. MF990558); +, positive (based on method used); –, negative (based on method used). †In addition to camel prion disease, a presumptive diagnosis of enterotoxemia had been made prior to death. Necropsy confirmed enterotoxemia, revealing digestive acidosis with hepatorenal degeneration. In dromedaries, this condition may manifest with neurological signs such as tremors, ataxia, aggression, hyperexcitability, convulsions, recumbency, opisthotonos, and death. ‡Animals P81/13 and P81/14 showed scattered vacuoles restricted to the basal ganglia; no spongiform alterations were observed in the cerebral cortices, medulla, or cerebellum.

**Figure 1:**
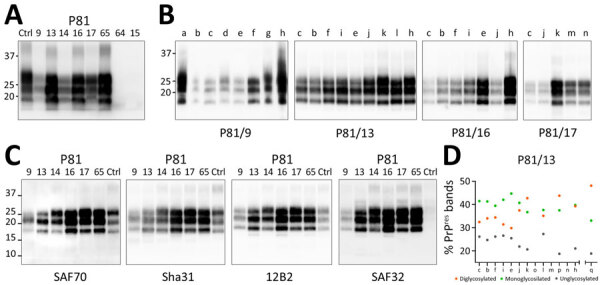
Detection and characterization of the proteinase K–resistant core (PrP^res^) of the pathological isoform of prion protein (PrP^Sc^) from brain tissues of dromedary camels, Tunisia, 2019–2021. A) PrP^res^ detected using TeSeE Western blot kit. Membrane probed with the Sha31 monoclonal antibody. Loading order (left to right): ovine scrapie control, Tunisian camel prion disease–positive cases (animal nos. P81/9–65), and Tunisian camel prion disease–negative samples (animal nos. P81/64 and P81/15). All samples, except for P81/64 and P81/15, were diluted 1:4 before electrophoresis and loaded at 3.75 mg tissue equivalent/lane. The negative samples were loaded at 15 mg tissue equivalent/lane. Approximate molecular weights (in kDa) are shown on the left side of the blot. B) Representative Western blot analysis of different available brain regions from selected positive cases. Brain tissues analyzed using the ISS discriminatory Western blot protocol. Case identifiers are indicated below each blot; corresponding brain regions are labeled at the top. All membranes were probed with the 12B2 antibody, except for P81/17, which was probed with L42. Tissue equivalents loaded per lane: 2 mg for P81/9 and 0.5 mg for P81/13, P81/16, and P81/17. Molecular weights (in kDa) are shown on the left side of the blot. C) Representative replica blots showing the epitope mapping analysis of PrP^res^ from brain homogenates of positive dromedary camel cases. A preliminary assessment of dromedary PrP^res^ reactivity with a panel of monoclonal antibodies was performed to identify suitable diagnostic tools for camel prion disease and to characterize PrP^res^. Within each antibody group, the antibody with the best sensitivity toward dromedary PrP^Sc^ was chosen for epitope mapping (Appendix Table 2, Figure 1). In addition, the SAF32 antibody, which targets the octarepeat region, was included to enable comparison with scrapie, in which that epitope is partially lost. Brain areas analyzed for each sample: P81/9, prefrontal cortex; P81/13, frontal cortex; P81/14, thalamus; P81/16, parietal cortex; P81/17, prefrontal cortex; P81/65, not identifiable; scrapie, medulla oblongata. A scrapie-affected sheep sample was included as control in last lane of each blot. Molecular weights (in kDa) are shown on the left side of the blot. Membranes were probed with monoclonal antibodies indicated below each blot. D) Relative proportion of diglycosylated, monoglycosylated, and unglycosylated PrP^res^ bands in each available brain region of animal P81/13. Scrapie (q) is shown on the right for comparison. Quantifications were performed on membrane probed with 12B2 antibody. Brain regions: a, striatum; b, frontal cortex; c, prefrontal cortex; d, capsule; e, occipital cortex; f, parietal cortex; g, cerebellar peduncle; h, cerebellum; i, temporal cortex; j, basal ganglia; k, thalamus; l, hippocampus; m, midbrain; n, medulla oblongata; o, hypothalamus; p, pons. Ctrl, control.

In addition to a histopathologic examination for spongiform changes, we examined all brains for the presence of the pathological isoform of prion protein (PrP^Sc^) by using Western blot (WB) and immunohistochemistry (IHC) (Appendix). We performed WB analyses by using the TeSeE western blot kit (Bio-Rad Laboratories, https://www.bio-rad.com) and the ISS discriminatory WB method, validated for the animal prion diseases surveillance in Europe. Six animals tested positive, revealing the presence of the pathognomonic protease-resistant PrP^Sc^ (PrP^res^), characterized by the classical 3-band pattern in the 18–30 kDa range, in all available brain regions (Appendix Table 1), providing evidence of the involvement of several brain areas (Table; Figure 1, panels A, B). Conversely, 2 animals tested negative.

After confirmation of PrP^Sc^ presence we conducted in-depth analysis of the 6 positive cases to investigate PrP^res^ features, including protease cleavage site, presence of additional fragments, and glycosylation pattern. We treated samples with high concentrations of proteinase K to clearly define the cleavage site and probed them with a panel of monoclonal antibodies spanning the PrP sequence (Appendix Table 2, Figure 1).

PrP^res^ from dromedary isolates displayed a higher apparent molecular weight of the unglycosylated band than the scrapie control, and we detected no additional C-terminal or internal fragments (Figure 1, panel C; Appendix Figure 1). PrP^res^ exhibited lower overall glycosylation levels compared with scrapie, mainly in cortical areas relative to subcortical regions (Figure 1, panel D; Appendix Figure 2). The PrP^res^ characteristics of the cases, including the electrophoretic profile, molecular weight and glycoprofile, are consistent with those previously reported in CPrD cases in Algeria (*1*).

We initially performed histopathologic and immunohistochemical analyses on the medulla oblongata and cerebellum, the primary target regions for animal TSE surveillance. We subsequently extended the analyses to other areas of the brains available (Appendix Table 1).

**Figure 2:**
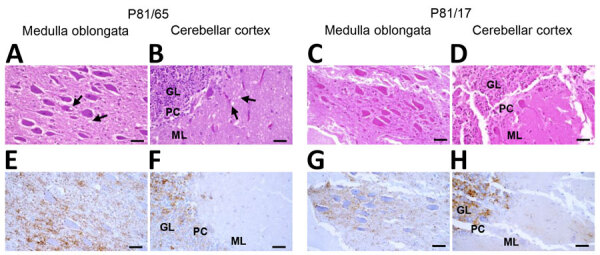
Histopathologic and immunohistochemical analyses of medulla oblongata and cerebellum of dromedary camels, Tunisia, 2019–2021. Images show the analyses performed on medulla oblongata (A, E, C, G) and cerebellum (B, F, D, H) of 2 representative animals (P81/65 and P81/17). Hematoxylin and eosin staining of the obex (A, C) and cerebellar cortex (B, D) revealed spongiform changes in the nucleus of the solitary tract (A, arrows) and in the molecular layer of cerebellar cortex (B, arrows) of P81/65, but no such alterations were observed in P81/17 (C, D). Conversely, immunohistochemistry revealed deposition of the pathological isoform of prion protein in both animals (E–H), affecting the nucleus of solitary tract (E, G) and cerebellar cortex (F and H). Brown color indicates PrP^Sc^ deposition. Brain sections from P81/17 showed partial loss of tissue integrity. Scale bars indicate 20 μm. GL, granular layer; ML, molecular layer; PC, Purkinje cells.

Histopathologic examination revealed spongiform changes in only 2 animals (P81/16 and P81/65), in both obex (mainly in the dorsal vagal nucleus and the nucleus of the solitary tract) and cerebellar cortex (molecular layer), whereas we observed no lesions in the other animals (Table; Figure 2, panels A–D). Immunohistochemistry performed on the medulla oblongata and cerebellum demonstrated the presence of PrP^Sc^ in all samples except P81/15 and P81/64. In the obex, we observed intense PrP^Sc^ immunoreactivity in the dorsal vagal nucleus, the nucleus of the solitary tract (Figure 2, panels E, G), the hypoglossal and olivary nuclei, and the reticular formation. In the cerebellum, we detected extensive PrP^Sc^ immunolabeling throughout all layers of the cerebellar cortex (Figure 2, panels F, H) and in the white matter.

**Figure 3:**
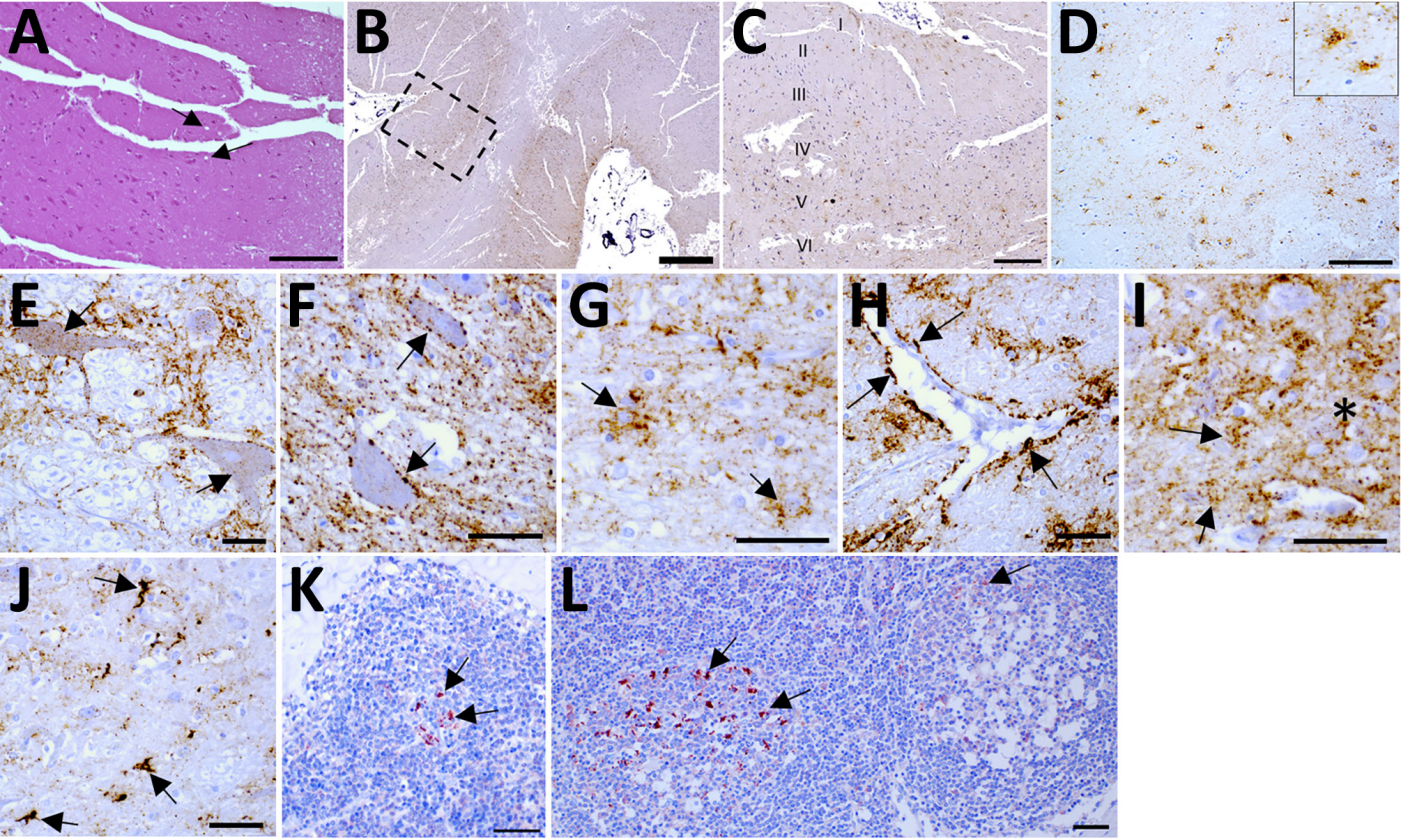
Histopathologic and immunohistochemical analyses of brain tissues and lymph nodes of camel prion disease–affected dromedary camels, Tunisia, 2019–2021. Examination of all available cerebral cortices (Appendix Table 3) showed mild spongiform changes exclusively in the temporal and occipital cortex (A) of sample P81/16. B) Immunostaining of the pathological isoform of prion protein (PrP^Sc^) found in prefrontal cortex of sample P81/13. C) Magnification of the prefrontal cortex (dashed box in panel B) showing the involvement of the I and V–VI layers. D) Intraglial (inset) and intraneuronal PrP^Sc^ depositions observed in the thalamus in animal P81/9. E–I) PrP^Sc^ deposition patterns observed in camel prion disease–affected brain tissues: intraneuronal (E, arrows), perineuronal (F, arrows), glial associated (G, arrows), perivascular (H, arrows), punctate (I, arrows) and diffuse (I, asterisk) in the neuropil. J) Dense intra-astrocytic PrP^Sc^ deposition (arrows) observed in the medulla oblongata. K–L) PrP^Sc^ deposition, observed in primary and secondary follicles of lymph nodes, appeared as a reticular network within the center of lymphoid follicles, accompanied by fine to coarse cytoplasmic granules in nonlymphoid cells. Representative immunostaining (arrows) of mandibular lymph node from animal P81/65 (K) and prescapular lymph node from animal P81/17 (L) highlights intense granular PrP^Sc^ depositions in tingible body macrophages within germinal centers. Brown indicates PrP^Sc^ deposition in brain sections; red indicates PrP^Sc^ deposition in lymph nodes. Scale bars indicate 50 µm in panels A and D, 100 µm in panel B, 250 µm in panel C, and 20 µm in panels E–L.

We extended histopathological and IHC analyses to other brain regions available for each case (Appendix Table 3). We observed mild spongiform changes exclusively in the cortices of animal P81/16 (Figure 3, panel A) and scattered vacuoles in the basal ganglia of P81/13 and P81/14.

IHC confirmed the diagnosis established from the medulla and cerebellum, identifying the same 6 CPrD-positive animals. In the cortex, PrP^Sc^ deposition was predominantly localized to layers I, V, and VI (Figure 3, panels B, C). The basal ganglia and thalamus (Figure 3, panel D) showed marked PrP^Sc^ accumulation in gray and white matter. We identified multiple PrP^Sc^ deposition patterns, including punctate and diffuse, intraneuronal, perineuronal, stellate, and perivascular (Figure 3, panels E–I). Of note, in the medulla oblongata, we observed a dense intra-astrocytic PrP^Sc^ deposition that filled the entire cytoplasm (Figure 3, panel J). IHC revealed PrP^Sc^ deposits in the primary and secondary follicles of all available lymph nodes, with variable immunostaining intensity (Table; Figure 3, panels K, L).

We conducted sequencing analysis of the entire PrP coding sequence. The analysis revealed that the 7 dromedary camels shared the same genetic background, being homozygous for the wild-type prion protein allele (Table).

## Conclusions

In this study, we report the detection of CPrD in dromedary camels in Tunisia, providing further evidence for the geographic distribution of the disease and supporting the hypothesis of its broader presence in North Africa. Analyses were concordant across methods; 6 of 8 suspected camels tested positive, confirming that standard tools (e.g., kits, WB, IHC, monoclonal antibodies) reliably detect PrP^Sc^ in CPrD and provide effective methods for diagnosis and surveillance.

The detection of PrP^Sc^ in all available lymph nodes, together with the relatively young age of some camels, supports the hypothesis that CPrD is a transmissible prion disease. As in scrapie and chronic wasting disease, lymphoid involvement may enable extraneural propagation and serve as a source of environmental contamination (*2*–*5*), raising concerns about prion shedding and persistence.

Despite the typically long incubation periods of prion diseases, several affected camels were relatively young, showing clinical signs at 3 years of age, when sexual maturity is only just reached (3–5 years). That finding might reflect early-life or high-dose exposure, given that extensive environmental contamination can shorten incubation (*6*,*7*). Similar patterns are reported in scrapie and chronic wasting disease, where vertical and early-life transmission contribute to infection in young animals (*4*,*8*–*14*).

The recent detection of sheep scrapie in the same area of Tunisia as CPrD (*15*) raises the question of possible links, although no similarities have yet been shown. Bioassays will be crucial to clarify strain relationships and whether environmental factors might facilitate cross-species transmission or adaptation.

Taken together, the data from Algeria and Tunisia suggest that CPrD may be endemic in certain areas. Despite the limited number of reported cases so far, the absence of active surveillance programs in those countries, unlike in Europe for scrapie and bovine spongiform encephalopathy (BSE), raises the possibility of a higher number of undetected cases. Given frequent cross-border movements and the extensive pastoral systems in which camels roam widely, contact between herds in Tunisia and herds in Algeria is frequent and has possible implications for disease circulation.

CPrD may substantially affect camel health and production, given that dromedaries are long-lived animals with extended reproductive and productive roles and are vital to pastoralist communities in arid regions. Its detection in areas where camels are central to food production underscores the need to understand its epidemiology, transmission, and long-term impact.

The emergence of a new animal prion disease also raises public health concerns. Although only BSE has shown zoonotic potential, its history highlights the necessity of monitoring all animal prion diseases. Long incubation and absence of early clinical markers complicate risk assessment, as exemplified by the decade-long delay between cattle BSE and the variant Creutzfeldt–Jakob disease epidemic in humans (*2*).

Given the potential implications for animal and human health and the possible socioeconomic impact in regions where dromedaries play a key role, ongoing surveillance and in-depth characterization of CPrD are essential to enhance our comprehension of its epidemiology, host range, and zoonotic potential.

Appendix Additional information about camel prion disease, Tataouine, Tunisia, 2019–2021.
