# Supplementary material for: Camel Prion Disease, Tataouine, Tunisia, 2019–2021
Source: Emerg Infect Dis. 2026 Aug;32(8):1350–5. doi: 10.3201/eid3208.251474 (PMC13426845; doi:10.3201/eid3208.251474)
Supplement: Appendix — Additional information about camel prion disease, Tataouine, Tunisia, 2019–2021. [file 25-1474-Techapp-s1.pdf]

Article DOI: https://doi.org/10.3201/eid3208.251474

EID cannot ensure accessibility for supplementary materials supplied by authors. Readers who have difficulty accessing supplementary content should contact the authors for assistance.

Camel Prion Disease, Tataouine, Tunisia, 2019–2021

Appendix

Materials and Methods

Animals and tissues

Over a period of approximately three years (2019-2021), eight dromedary camels (all the Maghrébi breed) displaying neurological signs compatible with the clinical presentation of CPrD, resulting negative for rabies, were reported (Table, https://wwwnc.cdc.gov/EID/article/32/8/25-1474-T1.htm). Some cases had been reported by the local breeders under the term "Medhbouba", denoting a neurological disorder in camels. The condition is initially characterized by disorientation of the animal, which ceases to follow the flock, and subsequently accompanied by neurological signs such as head swaying movements, hyperexcitability, teeth grinding and occasionally ataxia. All animals originated from the Tataouine governorate, except for one case from the Sousse governorate and one of Algerian origin but grazing in Tunisia and collected in the Tataouine governorate (Table). Both frozen and formalin-fixed brain tissues were collected from these animals (Appendix Table 1), although many samples showed evidence of tissue degradation related to storage and transportation conditions. Additionally, formalin-fixed lymph nodes were sampled from some animals: retropharyngeal lymph node (P81/9), mandibular lymph node (P81/17, P81/65), prescapular lymph node (P81/17), and unspecified lymph node from the head (P81/16).

Brain samples were screened for the presence of rabies virus using the Fluorescent Antibody Test (FAT) and the Rabies Tissue Culture Infection Test (RTCIT) at the Rabies Laboratory of the Pasteur Institute of Tunis.

Anti-Prion protein monoclonal antibodies

Several monoclonal antibodies (mAbs) with different epitopes were used for Western blot (WB) and immunohistochemistry (IHC): EP1802Y by Abcam (Cambridge CB2 0AX UK), SAF70, SAF84, SAF32, Sha31 were obtained by Bertin Pharma (78180 Montigny-le-Bretonneux, France), L42 and P4 by R-Biopharm (64297 Darmstadt, Germany), mAb132 by Creative Biolabs (NY 11967, USA), 9A2 and 12B2 by Wageningen Bioveterinary Research-WBWR (8221 RA Lelystad, The Netherlands).

PrP^{res} detection

The brain tissues were analysed by a commercially available Western blot test (TeSeE Western blot; Bio-Rad Laboratories, Inc., Hercules, CA, USA) and an in-house western blot protocol for the detection of the protease-resistant core of PrP^{Sc} (PrP^{res}). The western blot by TeSeE kit was performed as recommended by the manufacturer (Bio-Rad), using the monoclonal antibody Sha31 (AbI kit reagent). The in-house western blot diagnosis was made by a modified ISS discriminatory WB method (see below) that employed 50 µg/mL of Proteinase K (PK) and mAbs L42 and/or 12B2.

Genotyping of dromedary camel PRNP

DNA was extracted from 100 mg of frozen brain tissue with DNeasy Blood and Tissue Kit (QIAGEN, Hilden, Germany) following the manufacturer's instructions. The PrP gene (PRNP) coding sequence was amplified in a 50 µL final volume using 5 µL of extracted DNA, 1× AmpliTaq Gold 360 PCR Buffer (Applied Biosystems, Foster City, CA, USA), 2.5 mmol/L $MgCl_2$, 1× 360 GC Enhancer, 200 µmol/L dNTPs, 0.25 µmol/L of forward (5′-GCTGACACCCTCTTTATTTTGCAG-3′) and reverse (5′-GATTAAGAAGATAATGAAAACAGGAAG-3′) primers (*1*), and 0.5 µL of AmpliTaq Gold 360 (Applied Biosystems), using the following amplification protocol: 5 min at 96° C; 30s at 96° C, 15s at 57° C, 9s at 72° C for 40 cycles and 4 min at 72°C. Amplicons were purified with the Illustra ExoProStar 1-Step clean-up kit (GE Healthcare Life Sciences, Little Chalfont, UK). Sequencing reactions were obtained using the BigDye Terminator v1.1 Cycle Sequencing Kit, purified using BigDye XTerminator Purification Kit, and detected with the ABI PRISM 3130 apparatus (Applied Biosystems). Sequences were analysed with SeqScape version 4 (Applied Biosystems) and compared to the wild-type allele (GenBank accession no. MF990558).

For one sample (P81/64), frozen tissue arrived at the laboratory thawed and with advanced autolytic changes. The brain material was nonetheless processed for analysis, but PrP genotype could not be determined.

Neuropathologic and immunohistochemical analyses

Formalin-fixed brain and lymphoid samples were decontaminated with formic acid for 1 hour and then embedded in paraffin wax. Paraffin-embedded tissue blocks were cut at 5 μm for hematoxylin and eosin (H&E) staining and immunohistochemistry (IHC). IHC was performed as described previously (*2*). Briefly, we treated sections with 98% formic acid for 5 min, followed by autoclaving in citrate buffer for 5 min at 121°C. We then incubated sections with 6% normal goat serum (Vector Laboratories, Burlingame, CA, USA) in phosphate-buffered saline (PBS) for 60 min. We performed immunohistochemical detection of PrP^{Sc} with L42 monoclonal antibody (mAb) (R-Biopharm, Darmstadt, Germany) at 0.01 μg/mL in PBS overnight at 4°C. Sections were incubated with secondary biotinylated mouse antibody (Vector Laboratories), followed by ABC Complex (Vector Laboratories), and diaminobenzidine (Sigma-Aldrich, St. Louis, MO, USA) for brain tissues and AEC (3-amino-9-ethylcarbazole, Vector Laboratories) for lymphoid tissues. Sections were counterstained using Mayer's hematoxylin. Each run included positive and negative control sections.

Biochemical characterization of PrP^{res}

PrP^{res} characterization of the positive samples was performed using the ISS discriminatory Western blot method, a validated method for prion strain discrimination within the framework of the European TSE surveillance programme (*3*). This method employs high concentrations of PK for digestion, in contrast to the diagnostic WB previously applied in this study, which used 50 μg/ml PK. The protocol enables molecular typing of the protease-resistant PrP^{res} core through the combined use of monoclonal antibodies recognizing distinct epitopes across the PrP protein. Brain homogenates at 10% (wt/vol) in 100 mmol/L Tris-HCl (pH 7.4) 2% sarkosyl were incubated for 1 h at 37°C with PK (Sigma-Aldrich, St. Louis, Missouri, USA) to a final concentration of 200 μg/mL. Protease treatment was stopped with 6 mmol/L PMSF (Sigma-Aldrich). Aliquots of samples were added with an equal volume of isopropanol/butanol (1:1 vol/vol) and centrifuged at 20,000 × g for 10 min. The pellets were resuspended in denaturing sample buffer (NuPAGE LDS Sample Buffer; Life Technologies) and heated for 10 min at 95°C. We loaded each sample onto 12% bis-Tris polyacrylamide gels (Invitrogen) for electrophoresis

with subsequent WB on polyvinylidene fluoride membranes using the Trans-Blot Turbo Transfer System (Bio-Rad) according to the manufacturer's instructions. The blots were processed with several anti-PrP mAbs by using the SNAP i.d. 2.0 system (Millipore, Burlington, MA, USA) according to the manufacturer's instructions. After incubation with the secondary antibody horseradish peroxidase-conjugated (HRP) anti-mouse immunoglobulin (Pierce Biotechnology, Waltham, MA, USA) at 1:20,000 (or goat anti-rabbit IgG (H+L) HRP at 1:10,000, G21234 Thermofisher Scientific, when we used EP1802Y as primary antibody), the PrP bands were detected by using enhanced chemiluminescent substrate (SuperSignal Femto; Pierce Biotechnology) and ChemiDoc imaging system (Bio-Rad). The chemiluminescence signal was quantified by using Image Lab 6.1.0 (Bio-Rad).

References

1. Kaluz S, Kaluzova M, Flint APF. Sequencing analysis of prion genes from red deer and camel. Gene. 1997;199:283–6. PubMed https://doi.org/10.1016/S0378-1119(97)00382-X

2. Babelhadj B, Di Bari MA, Pirisinu L, Chiappini B, Gaouar SBS, Riccardi G, et al. Prion disease in dromedary camels, Algeria. Emerg Infect Dis. 2018;24:1029–36. PubMed https://doi.org/10.3201/eid2406.172007

3. European Union Reference Laboratory for Transmissible Spongiform Encephalopathies. TSE strain characterisation in small ruminants. 2024 Jul [cited 2025 Sep 12]. https://www.eurl-tse.eu/wp-content/uploads/2024/09/EURL_smallruminants_discriminatory_guidance_v3_revised.pdf

4. Doolan KM, Colby DW. Conformation-dependent epitopes recognized by prion protein antibodies probed using mutational scanning and deep sequencing. J Mol Biol. 2015;427:328–40. PubMed https://doi.org/10.1016/j.jmb.2014.10.024

Appendix Table 1. Collected samples

	P81/9		P81/13		P81/14		P81/15		P81/16		P81/17		P81/64		P81/65	
Brain area	FF	F	FF	F	FF	F	FF	F	FF	F	FF	F	FF	F*	FF	F*
Prefrontal cortex		X	X	X	X		X			X		X				
Frontal cortex		X	X	X	X	X	X	X		X						
Parietal cortex	X	X		X		X				X						
Temporal cortex	X			X		X	X		X	X						
Occipital cortex	X	X		X					X	X	X		X			
Basal ganglia	X	X	X	X	X	X	X			X		X				
Thalamus	X			X		X	X					X				
Hypothalamus						X										
Hippocampus				X		X										
Midbrain						X						X				
Pons	X			X			X									
Medulla oblongata	X		X		X		X	X	X		X	X	X		X	
Cerebellum	X	X	X	X	X	X	X		X	X	X		X		X	

FF: formalin-fixed; F: frozen
* Unidentifiable areas: tissue received thawed and exhibiting autolytic changes. The brain material was nonetheless processed for analysis.

Appendix Table 2. Affinity of monoclonal antibodies for dromedary camel PrP^{Sc}

Antibody group	Antibody	Epitope*	Affinity†
N-terminus	SAF32	Octarepeat	+
	12B2	$_{92}$WGQGG$_{96}$	+++
	P4	Dr $_{92}$WGQGG$\underline{GA}$H$_{99;}$ Sh $_{93}$WGQGG-SH$_{99}$	-
Core	9A2	$_{102}$WNK$_{104}$	+++
	mAb132	$_{123}$AVVGGLGGY$_{131}$	+
	L42	$_{148}$YEDERYY$_{153}$	++
	Sha31	$_{148}$YEDRYYRE$_{155}$	+++
C-terminus	SAF70	$_{159}$RYPNQVY$_{165}$	++
	SAF84	Dr $_{166}$Y$\underline{K}$PVDQY$_{172;}$ Sh $_{166}$YRPVDQY$_{172}$	+/-
	EP1802Y‡	Dr $_{225}\underline{Y}$QA$_{227}$ and Y$_{229}$ Sh $_{225}$SQA227 and Y$_{229}$	-

* Dromedary prion protein (PrP) numbering. Whenever a substitution is present, both dromedary (Dr) and sheep (Sh) PrP sequences are reported, and sequence variations are underlined
† Affinity based on the last detectable dilution of Tunisian CPrD.
‡ Epitope as defined by Doolan and colleagues (*4*). Notably, all antibodies that showed weak or no detection of dromedary PrP target epitopes correspond to regions with sequence variation in the dromedary PrP protein.

Appendix Table 3. Results of histopathological and immunohistochemical analyses.

	P81/9		P81/13		P81/14		P81/15		P81/16		P81/17		P81/64		P81/65	
Brain area	H&E	IHC	H&E	IHC	H&E	IHC	H&E	IHC	H&E	IHC	H&E	IHC	H&E	IHC	H&E	IHC
Prefrontal cortex			-	+	-	+	-	-								
Frontal cortex			-	+	-	+	-	-								
Parietal cortex	-	+														
Temporal cortex	-	+					-	-	+	+						
Occipital cortex	-	+							+	+	-	+	-	-		
Basal ganglia	-	+	+	+	+	+	-	-								
Thalamus	-	+					-	-								
Pons	-	+					-	-								
Medulla oblongata	-	+	-	+	-	+	-	-	+	+	-	+	-	-	+	+
Cerebellum	-	+	-	+	-	+	-	-	+	+	-	+	-	-	+	+

H&E = hematoxylin and eosin staining; IHC = immunohistochemistry; +/- = resulted positive/negative based on method used

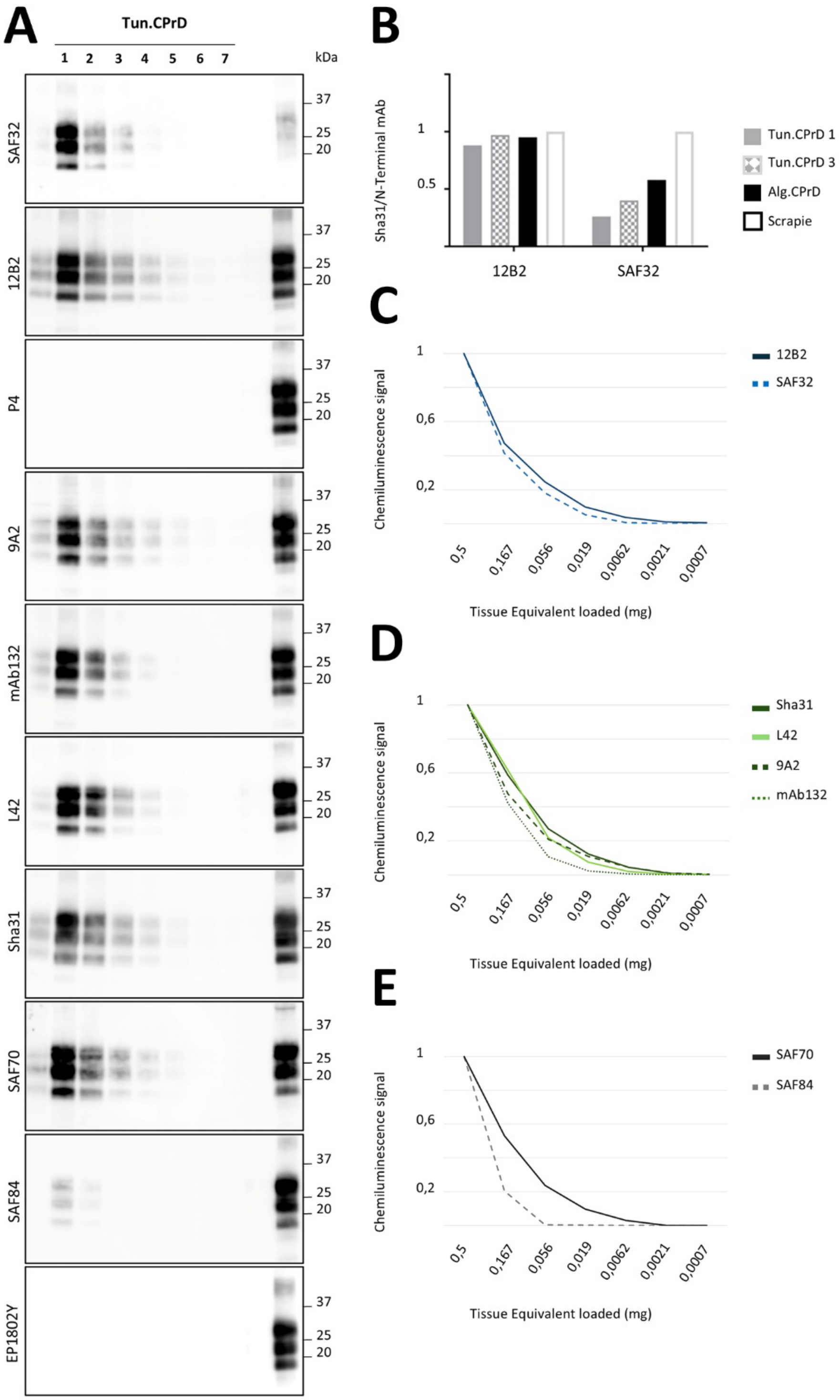

Appendix Figure 1. Western blot analysis of Tunisian and Algerian CPrD and evaluation of mAbs sensitivity. A preliminary assessment of dromedary PrP^{res} reactivity with a panel of monoclonal antibodies was performed to identify suitable diagnostic tools for CPrD and to characterize PrP^{res}. Within each antibody group, the antibody with the best sensitivity toward dromedary PrP^{Sc} was chosen for the subsequent epitope mapping analysis. A) Representative western blot of PK-treated (50 µg/ml) brain

homogenate of dromedary camel CPrD isolates from Algeria (Alg. CPrD) (*2*) and Tunisia (Tun. CPrD), and sheep classical scrapie. Replica blots were probed with different monoclonal antibodies (mAbs), as indicated on the left of each blot. The Tunisian CPrD sample was subjected to a 1:3 serial dilution (indicated as 1 to 7 in the blots). Tissue equivalents (TE) loaded per lane were 0.9 mg for Alg. CPrD, 0.5 mg for the first dilution point of the Tunisian CPrD (indicated as 1), and 0.5 mg for classical scrapie. The molecular weights (expressed in kDa) are indicated on the right of each blot. Based on the titration results, mAbs used in epitope mapping can be divided into 4 different groups based on the ability to detect PrP^{res} (Appendix Table 2): +/-) SAF84, which detects CPrD PrP^{res} only until the 2nd dilution (0.167 mg TE loaded); +) SAF32 and mAb132 able to detect until the $3^{rd}/4^{th}$ dilution (between 0.056 mg and 0.019 mg TE loaded); ++) SAF70 and L42 which detect until the $4^{th}/5^{th}$ dilution (between 0.019 mg and 0.0062 mg TE loaded); +++) Sha31, 9A2 and 12B2 which can detect until the $5^{th}/6^{th}$ dilution (between 0.0062 mg and 0.0021 mg TE loaded). Note that the Algerian CPrD sample is characterised by an extremely low amount of PrP^{Sc}, comparable to the amount detected in the third dilution of the Tunisian CPrD sample. B) Graph depicting the antibody ratio (Sha31/N-Terminal Ab) of Alg. CPrD, undiluted Tun. CPrD (ID 1 in the blots in panel A), and Tun. CPrD diluted 1:9 (ID 3) relative to the Sha31/N-terminal Ab ratio of control scrapie. The ratio using 12B2 as N-terminal mAb is reported on the left, while using SAF32 on the right. The Sha31/12B2 ratio showed values close to 1 for all the samples, indicating the conservation of the 12B2 epitope in CPrD PrP^{res}. Importantly, the Sha31/SAF32 ratio was extremely low, thus indicating that the SAF32 epitope is preserved upon PK cleavage in CPrD while being partially cleaved in classical scrapie. C, D, E) Graphs depicting the chemiluminescence signal intensity obtained with antibodies used in panel A, across serial dilutions of the Tunisian CPrD sample. The y-axis represents the ratio of the chemiluminescence signal at each dilution relative to the signal at the first dilution, which is set as 1. The x-axis represents the tissue equivalents loaded (expressed in mg) in each dilution. The antibodies are grouped based on their epitope location on PrP, i.e., N-terminal region (C), core region (D), and C-terminal region (E). Among the antibodies targeting the N-terminal region of PrP (C), SAF32 shows a rapid signal decline and lower ratio values compared to 12B2 up to the 4th dilution, where its signal tends to zero. In contrast, 12B2 still maintains a detectable signal up to the 6th dilution. D) In line with the qualitative classification based on the last detectable dilutions (A) and similarly to mAb 12B2, antibodies 9A2 and Sha31 maintain a detectable signal at extreme high dilutions, in contrast to mAb132 and L42, for which the ratios tend to zero at the 4th and 5th dilution, respectively. Note that, despite 9A2 shows a more rapid signal decline than L42 in the first dilutions, an inversion of the chemiluminescence ratio of the two antibodies is observed from the 4th dilution onwards. Finally, among mAbs targeting the C-terminal region of PrP (E), SAF84 signal dramatically decreased at the second dilution point, dropping to nearly 0 at the third dilution, in contrast to SAF70, whose chemiluminescence ratio tends to zero only at the 5th dilution.

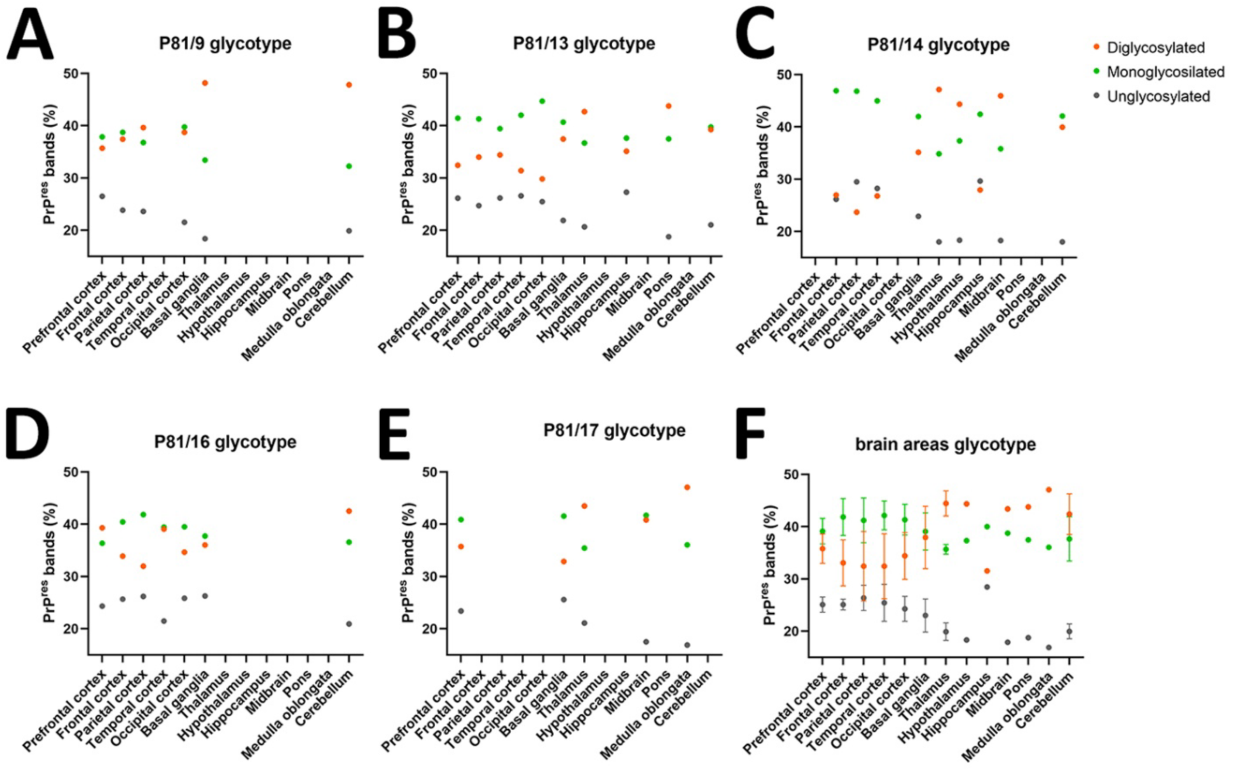

Appendix Figure 2. Analysis of glycotype of PrP^{res}. Graphs show the relative proportion of di-, mono-, and unglycosylated PrP^{res} bands in each available brain region of positive dromedary camels (A-E). The last graph (F) shows the mean and standard deviation of PrP^{res} forms from all animals in each brain region. Quantifications were performed on membranes probed with 12B2 mAb. Analysis of all available brain regions revealed minor variability; however, a consistent pattern was observed across all positive cases, with reduced PrP^{res} glycosylation in cortical areas relative to subcortical regions. Notably, the monoglycosylated isoform predominated over the diglycosylated form in the cortex. For some brain areas, samples from at least three animals were not available to calculate mean values and standard deviations. For areas available from only one animal, individual values are shown: hypothalamus (P81/14), pons (P81/13), and medulla oblongata (P81/17). For areas available from two animals, the mean value is reported: hippocampus (P81/13 and P81/14) and midbrain (P81/14 and P81/17).
